# Implementation of integrated care for diabetes mellitus type 2 by two Dutch care groups: a case study

**DOI:** 10.1186/s12875-015-0320-z

**Published:** 2015-08-21

**Authors:** Loraine Busetto, Katrien Luijkx, Anna Huizing, Bert Vrijhoef

**Affiliations:** Tranzo Scientific Center for Care and Welfare, Tilburg University, Warandelaan 2, 5037 AB Tilburg, The Netherlands; Department of Family Medicine, Maastricht University, Minderbroedersberg 4-6, 6211 LK Maastricht, The Netherlands

**Keywords:** Integrated care, Chronic care, Diabetes, Implementation, CMO Model, Chronic care model, Implementation model

## Abstract

**Background:**

Even though previous research has demonstrated improved outcomes of integrated care initiatives, it is not clear why and when integrated care works. This study aims to contribute to filling this knowledge gap by examining the implementation of integrated care for type 2 diabetes by two Dutch care groups.

**Methods:**

An embedded single case study was conducted including 26 interviews with management staff, care purchasers and health professionals. The Context + Mechanism = Outcome Model was used to study the relationship between context factors, mechanisms and outcomes. Dutch integrated care involves care groups, bundled payments, patient involvement, health professional cooperation and task substitution, evidence-based care protocols and a shared clinical information system. Community involvement is not (yet) part of Dutch integrated care.

**Results:**

Barriers to the implementation of integrated care included insufficient integration between the patient databases, decreased earnings for some health professionals, patients’ insufficient medical and policy-making expertise, resistance by general practitioner assistants due to perceived competition, too much care provided by practice nurses instead of general practitioners and the funding system incentivising the provision of care exactly as described in the care protocols. Facilitators included performance monitoring via the care chain information system, increased earnings for some health professionals, increased focus on self-management, innovators in primary and secondary care, diabetes nurses acting as integrators and financial incentives for guideline adherence. Economic and political context and health IT-related barriers were discussed as the most problematic areas of integrated care implementation. The implementation of integrated care led to improved communication and cooperation but also to insufficient and unnecessary care provision and deteriorated preconditions for person-centred care.

**Conclusions:**

Dutch integrated diabetes care is still a work in progress, in the academic and the practice setting. This makes it difficult to establish whether overall quality of care has improved. Future efforts should focus on areas that this study found to be problematic or to not have received enough attention yet. Increased efforts are needed to improve the interoperability of the patient databases and to keep the negative consequences of the bundled payment system in check. Moreover, patient and community involvement should be incorporated.

## Background

Previous research has shown that integrated care initiatives can lead to improved processes and patient outcomes [1–4]. However, this is not always the case and there is a lack of evidence regarding the reasons why and in which cases integrated care works [5, 6]. Consequently, researchers have called for increased emphasis on the implementation process and its relationship to outcomes [7–9]. To this purpose, Pawson and Tilly suggest the “Context + Mechanism = Outcome Model” (CMO Model), stipulating that interventions only have successful outcomes when they introduce appropriate mechanisms in appropriate contexts [10].

Two recent literature reviews examining 44 studies on the implementation of integrated care for diabetes attempted to analyse the effectiveness of integrated care in light of the CMO Model (Busetto, L., Luijkx, K.G., Elissen, A.M.J., Vrijhoef, H.J.M., unpublished). These reviews found that most integrated care interventions included all components of the Chronic Care Model (CCM) and reported improved patient, process and health service utilisation measures. Moreover, most barriers were related to the organisational context, while most facilitators were related to the social context. However, given the lack of comparable outcome measures as well as in depth qualitative data, it was not possible to make statements about the relationship between context, mechanisms and outcomes. It was suggested that more research with the CMO Model in mind is needed. This study aims to contribute to filling this knowledge gap by conducting research on the context, mechanisms and outcomes of integrated care for type 2 diabetes.

This study is part of Project INTEGRATE, financed by the European Commission, studying geriatric conditions, chronic obstructive pulmonary disease (COPD), mental health problems and type 2 diabetes. The current study describes a case study on two care groups providing integrated care for people with type 2 diabetes in the Netherlands and aims find out how mechanisms and context have influenced the outcomes of integrated care for type 2 diabetes as implemented by the two care groups. Care groups are legal entities that establish contracts with health insurers and health professionals in order to coordinate the so-called ‘care chain’ of chronic care from diagnosis to after care [11]. Integrated care for chronic conditions has been in the process of development in the Netherlands since the 1980s. Today, integrated care is characterised by care groups and the bundled payment system, patient involvement, health professional cooperation and task substitution, evidence-based care protocols and the use of a shared clinical information system (see Table 1).

**Table 1:** Core elements of integrated care for type 2 diabetes in the Netherlands

Element	Description
Care groups and bundled payment system	Dutch integrated care for type 2 diabetes is organised via so-called care groups, legal entities that “establish contracts with health insurers in order to coordinate and execute chronic care in a specified region, with the aim of improving the quality of care” [11]. The legal form of these organisations varies but, in most cases, general practitioners (GPs) are (co-) owners [24]. In 2010, there were approximately 100 care groups offering integrated care for diabetes, many of which were also offering programmes for other chronic conditions such as COPD and vascular risk management [24]. In the funding framework introduced in 2007, the so-called bundled payment system, care groups act as intermediaries between health insurers and health care professionals by negotiating the content and price of a comprehensive package of diabetes care, the resulting agreements of which are captured in bundled payment contracts [24]. These contracts make it possible to buy care as if it were one single product, even though it consists of many components delivered by a diverse group of health care professionals often in more than one setting [24]. For those health care services not provided by GPs and practice nurses (PNs), care groups enter contracts with care chain partners such as dieticians, podiatrists or pedicurists, depending on the chronic condition.
Evidence-based care protocols	In the Netherlands, care provision for type 2 diabetes is based on national evidence-based care standards describing norms of high quality chronic care for specific chronic diseases, such as the diabetes care standard [23]. Based on the negotiations between care groups, health care professionals and health insurers, these standards are translated into specific care protocols, based on which, care is delivered and reimbursed. Negotiations do not only take place between one care group and one health insurer or one care group and one health care professional, but each care group enters into contracts with potentially all health insurers and all relevant health care professionals in a given region and vice versa [29, 30].
Health professional cooperation and task substitution	The delivery of diabetes care is performed by a group of health care professionals involved in the care for a specific chronic disease. The core of diabetes care includes GPs, PNs, diabetes nurse specialists (DNSs) and internists. The former two are located in general practice, whereas internists are located at the hospital, and DNSs are dispatched from hospital to general practice and are therefore present at both locations [31, 32]. It should be noted that, while internists are involved in the provision of integrated diabetes care, whether they are reimbursed via the bundled payment contract differs per region and care group. At the periphery, dieticians, podiatrists, pedicurists, optometrists and other medical specialists are also involved [23, 24]. Dutch integrated care is based on the assumption that substitution of professional roles and tasks will lead to more cost-efficient care. Horizontal substitution means the transition of patients from secondary to primary care, where stable diabetes patients as a default should be treated by the GP instead of the internist. Vertical substitution means that certain tasks traditionally performed by the GPs or internists are now performed by PNs and DNSs, respectively [11, 32].
Patient involvement	Involvement of patients both during consultations and in the organisation of health care is an important strategy in the Dutch approach to integrated care for type 2 diabetes [23]. One example of the former is shared patient-doctor goal-setting, mostly realised via individual care plans which, as opposed to general treatment plans, consist of precise and feasible goals that are set in a shared-decision making process between patient and health care professional. An approach to involving patients in the organisation of diabetes care is the consultation with patient advisory boards. With Dutch integrated care still being in development, both aspects of patient involvement are not yet fully implemented in practice [24].
Shared clinical information system	The electronic administration and exchange of data for patients with type 2 diabetes treated within the bundled payment framework is an important requirement for integrated care [23]. However, the number and type of electronic databases used in practice differs per region and care group. Often, GPs use their own GP information system (HIS) and practice nurses and care chain partners use a care chain information system (KIS). Care chain partners also use their own profession-specific electronic medical record systems. The HISs are commercial systems that can be chosen freely according to the GP’s own preferences. A KIS, on the other hand, is generally chosen by the care group, and every GP practice and care chain partner working with a certain care group must work with this specific KIS [24].

The remainder of the article will outline the study methods, present and discuss the study’s findings and end with a conclusion including recommendations for further research.

## Methods

This study adopts an embedded single case design with two units of analysis. This case study is based on a protocol described in detail elsewhere [12].

### Case selection

The two care groups invited to participate were selected as best practices based on the following criteria: nomination as national best practices by leading health research institutions, participation in previous (diabetes) research, and involvement in care innovation pilots [12, 13]. Best practices research is a popular approach but controversial, mostly due to problematic external validity [14, 15]. However, focusing on best practices is expected to generate an important potential for learning by other Dutch care groups, and, given the Netherlands’ long experience in integrated care and status as a pioneer, also for other European and non-European countries [16, 17].

### Concepts

We distinguished between context, mechanisms and outcomes according to the CMO Model which states that interventions have successful outcomes only when they are introduced by appropriate mechanisms in the appropriate context [10, 18, 19].

‘Context’ was understood as the setting in which the mechanisms are brought into practice and described by outlining the barriers and facilitators to change encountered in the implementation process. Barriers and facilitators were categorised and analysed using the Implementation Model (IM) by Grol and Wensing, which specifies six levels of health care at which barriers and facilitators to change can occur, *i.e.* innovation (advantages in practice, feasibility, credibility, accessibility, attractiveness), individual professional (awareness, knowledge, attitude, motivation to change, behavioural routines), patient (knowledge, skills, attitude, compliance), social context (opinion of colleagues, culture of the network, collaboration, leadership), organisational context (organisation of care processes, staff, capacities, resources, structures) and economic and political context (financial arrangements, regulations, policies) [20].

‘Mechanisms’ were defined as the different elements of integrated care and categorised according to the CCM by Wagner [21]. The CCM states that improvements in integrated care for chronic conditions require changes in six components (health system, self-management support, delivery system design, decision support, clinical information system and community [22]). Generally, interventions targeting at least two of these components are considered integrated care [1, 2, 4]. As indicated earlier, Dutch integrated care is characterised by care groups and the bundled payment system, patient involvement, health professional cooperation and task substitution, evidence-based care protocols and the use of a shared clinical information system (see Table 1). We mapped these elements to five of the six CCM components as shown in Table 2.

**Table 2:** Dutch integrated care for type 2 diabetes by CCM components

Dutch integrated care for type 2 diabetes	CCM component
Care groups and bundled payment system	Health system
Patient involvement	Self-management support
Health professional cooperation and task substitution	Delivery system design
Evidence-based care protocols	Decision support
Shared clinical information system	Clinical information system
	Community

No aspects of integrated care relating to the community component (*i.e.* mobilising community resources to meet the needs of patients) have been found in the relevant literature and documents, including national diabetes care standards and a previous major evaluation of current Dutch integrated care [23, 24].

‘Outcomes’ were defined as the effects triggered by mechanism and context. In our analysis, we used quality of care as the main outcome variable as this was the type of outcome discussed most frequently by the interviewees. Our understanding of quality of care was informed by the WHO’s operational definition spanning six dimensions of quality including effective, efficient, accessible, patient-centred, equitable and safe health care [25].

### Data collection

Data were collected from the case sites by means of semi-structured interviews. Initial contact between research team and interviewees was established via the case sites’ contact persons. The response rate was 96 %. In total 26 interviews (13 per care group) were conducted between May 2013 and January 2014 with care group managers and staff, care purchasers as well as health care professionals such as general practitioners (GPs), internists, diabetes nurse specialists (DNSs), practice nurses (PNs), dieticians, optometrists, podiatrists, pedicurists and pharmacists. All interviewees signed an informed consent form, and all interviews were audio-taped and transcribed verbatim. The interviewers used a topic list to steer the conversation. Member checks were performed by sending a one-page summary to each interviewee to confirm the interpretation of their statements. In a reminder, interviewees were informed that a lack of response would be interpreted as agreement with the summary. Nineteen interviewees replied to the member check, of which 13 confirmed the summary without comment (or only minor textual adjustments) and six commented on the member check or provided an adapted version. These comments and changes were taken up in the analysis of the interview results.

Dutch law does not require medical or ethical reviews for interviews with health care professionals. Confidentiality was ensured by not disclosing the names or regions of the care groups and only referring to the interviewees by their functions.

### Data analysis

We chose a case-oriented approach to data analysis because of its suitability to studying “complex, context-bound, or context-sensitive” phenomena [26]. Using Atlas.ti 6, a sub-sample of the interview-transcripts was coded independently by two researchers (LB, KL) and the rest by one researcher (LB). A coding list was based on the conceptual models and topic list and further adapted in an iterative coding process. To ensure a homogeneous interpretation of interviews, a content check was performed for a heterogeneous sub-sample of interviews. Two researchers (KL, BV) checked ten interviews independently and compared them to the preliminary results provided by a third researcher (LB). The researchers (LB, KL, BV) discussed the results together until consensus was reached.

## Results

Table 3 presents an overview of the barriers and facilitators per IM level. In the following section, we present the results for each CCM component and IM level to adequately reflect the complex relationships between context, mechanisms and outcomes. For each section, we present the relationships between the CCM component, barriers and facilitators, and outcomes as explained by the interviewees.

**Table 3:** Overview of barriers and facilitators to the implementation of integrated care per IM level

IM level	Barriers	Facilitators
Innovation	• Disease-specific care management	• Performance monitoring via the care chain information system
	• Insufficient integration between the various patient databases	
Individual professional	• Decreased earnings	• Increased earnings
	• Too many innovations	• GP support
	• Resistance by GPs	
Patient	• Patients’ insufficient medical and policy-making expertise	• Increased focus on self-management
Social context	• Resistance by GP assistants due to perceived competition	• Innovators in primary and secondary care
		• Tradition of transmural cooperation
Organisational context	• Lack of qualified PNs	• Care group management and support
	• Too much care provided by PNs	• PNs and DNSs acting as integrators
Economic and political context	• The negative role of some health insurers	• Financial incentives for care innovations
	• Yearly changes in insurance policies	• Health insurer cooperation
	• The funding system incentivising the provision of care exactly as described in the care protocols	• Financial pressure in the health sector
		• Financial incentives for guideline adherence

In general, the economic and political context barriers were the topics discussed most saliently as the most problematic areas for implementing integrated care, followed by barriers relating to the patient databases. Patient involvement received very little attention, and community involvement was mentioned just once and only as a goal for future efforts.

### Health system

#### Individual professional

Interviewees reported that earnings of some care chain partners such as the dieticians, podiatrists and pedicurists had decreased with the introduction of the new funding system, which led to dissatisfaction among these groups. On the other hand, three interviewees indicated that integrated care had brought financial benefits for health care professionals, including certain care chain partners but especially GPs, and that this led to increased provider satisfaction.

Six interviewees indicated that when too many innovations were introduced too fast, health care professionals became tired and reluctant to implement changes. Care group staff were aware of this danger:

*“I think we’re less good at maintaining what’s there because we are so focussed on new developments and innovating all the time that we forget that we have a basis who we continually burden with everything we introduce and that for this some kind of support is very important and this should sometimes be at the expense of the time you invest in new developments”.* Care group B, staff

#### Organisational context

Many interviewees pointed out the pivotal role of the care group in supporting the health care professionals. It was also mentioned that it was helpful that the care group had multiple mutual obligations with the health insurer because these interdependencies increased the pressure for both parties to reach mutually acceptable agreements. Moreover, higher quality of care was facilitated by the existence of certain quality requirements which care chain partners had to fulfil to begin cooperating with a care group. Finally, the care purchasers pointed out that the care group was a helpful access point to introduce new innovations via established channels.

#### Economic and political context

Eight interviewees pointed out the dominant, powerful and sometimes even obstructive role of the health insurers during the negotiations with the care groups. One interviewee lamented that health care professionals were trying to improve the quality of care in spite of the health insurers, as a result of the health insurers’ focus on keeping the costs of care as low as possible. Many interviewees indicated that health insurers differed considerably with regard to their focus on costs vis-à-vis quality of care, and all interviewees who saw this difference said that health insurer X was also interested in quality, while health insurer Y tended to focus mainly on costs. This impression was confirmed by the purchasers from health insurer X.

Related to this is the major barrier that the Dutch insurance law allows people to choose new health insurance every year. Consequently, every year health insurers determine a new policy of what they are willing to finance. The frequency of these changes caused a lot of frustration in the care group and among the health care professionals:

*“We have to negotiate the price for our efforts every year. Every year again. It’s crazy in the long run and a lot of wasted energy. It’s terrible (…). The moment you sign an agreement you’re basically already negotiating for the next year”.* Care group B, GP

Despite the care group’s efforts it was not always possible to implement all changes before 1 January, the date on which new insurance policies take effect. This caused additional confusion and uncertainty because care was already provided while there were no definitive contracts and reimbursement rates were still unknown.

Three interviewees explained that the current funding system involved financial incentives to provide care exactly as described in the protocols. However, at times this meant that patients received too much or too little care. Several interviewees worried that this financing based on evidence-based care protocols might have been taken too far and now constituted a barrier to person-centred and high quality care.

On the upside, interviewees appreciated that the new funding system included financial incentives for new developments such as the care chain information system, online patient platforms, patient education courses, individualised health care and provider education. Moreover, it was pointed out that financial incentives to provide care as described in evidence-based care protocols led to a more efficient care delivery and higher overall quality of care. Five interviewees pointed out the role of one particular health insurer as a facilitating one. Care group A’s director explained that the care group had a very good working relationship with health insurer X. Interviewees also pointed out that the financial pressure in the national health sector made cooperation more urgent and therefore easier to accomplish, amongst other things, because financial constraints motivated people to become more innovative.

### Self-management support

#### Patient

Two interviewees mentioned an increased focus on self-management and a change in health care professionals’ attitudes towards more patient-centeredness:

“*And the most important thing, I believe, is what changed towards the patient, that the health care professionals started to realise that if the patient is not motivated, you will never ever succeed. (…) The idea that you can leave the patient just as he is and then you have to try to limit the damage with some kind of chemical warfare, that’s just… You need patient participation”.* Care group A, specialist

Both care groups indicated that there were attempts and experiences regarding structural patient involvement in the organisation of care but often patients were only involved on an incidental basis. Care group A used to involve patient representatives in the development of care programmes but this ended because patients themselves felt that the working groups required a level of policy making and medical expertise they did not have. Care group B structurally involves a patient advisory board in certain trajectories and developments of new projects. However, one interviewee pointed out that, while she thought that co-consultation with the patients was ensured, she doubted whether there was actual co-decision making as the patient advisory board was not represented in the body making the final decisions.

### Delivery system design

#### Innovation

At the level of the innovation itself, several interviewees indicated that, with so many diabetes patients suffering from multi-morbidity, disease-specific care management sometimes caused problems in practice. For example, care group A’s PN sometimes had to refer patients to the GP or a different PN for non-diabetes related questions. This resulted in confusion in the patients and the need for more complex planning by the PNs. Moreover, health care professionals were uncertain about data administration and invoicing when patients suffered from more than one medical condition.

#### Individual professional

When integrated care was first implemented, many GPs resisted its introduction because of the perceived loss of autonomy resulting from becoming part of a care group, which involved relatively strict care protocols, internist-supervision, PNs taking over a large part of diabetes care and increased transparency due to performance monitoring. Moreover, new developments around integrated care required the GPs to become managers of their own enterprises and to manage an increasing number of staff, for several of whom they became financially responsible. Not all GPs were equally capable or willing to assume this extended role.

Still, it was important for GPs to be convinced that integrated care would improve quality of care in order for them to become supportive of the integrated care intervention. It was also important for them to recognise the DNSs’ expertise which helped them to accept the DNs as partners. This also led the GPs to recommend the DNs to their colleagues.

#### Social context

When PNs were first introduced in general practice, the GPs’ assistants perceived them as a threat because they feared that PNs would take tasks away from them. However, eventually they realised that PNs took on new tasks relating to the management of chronically ill patients, while the assistants kept providing administrative support to the GP’s practice.

Interviewees from both care groups pointed out innovators in the primary as well as secondary care sector who initiated and advanced the implementation of integrated care in the region. Several interviewees reported that also after this initial phase, there was always a group of innovators to drive the developments. Five interviewees from region A mentioned the rich and longstanding tradition of transmural cooperation in the region which resulted in a good basis for building and further developing integrated care.

#### Organisational context

When the expanded role of the PN was introduced, it was difficult to find a sufficient number of qualified personnel. Until a structured training programme could be set up, personnel were enticed away from home care organisations. Two interviewees expressed their worry that, today, the substitution of care from GP to PN might have been taken too far and that now too much care was provided by the PN instead of the GP which impeded the quality of care provided.

By facilitating the cooperation between the GPs and care chain partners, both the PNs and the DNSs were reported as important actors in a truly integrated approach to health care provision and helped to ensure increased continuity of care for the patients. It was also pointed out that knowing the other partners personally and knowing everyone’s contribution to the patient’s care, improved cooperation. Mostly, these personal contacts were organised between the PN and the other health care professionals.

#### Economic and political context

In region A, four interviewees specifically emphasised the good atmosphere of cooperation between GPs and medical specialists across care sectors. One important reason for this good cooperation was thought to be the fact that the specialists were employed at an academic hospital where specialists receive a regular salary independent of the number of patients treated, as opposed to regular hospitals with a fee-for-service structure. This was assumed to remove financial competition from the relationship when the number of patients treated in primary instead of secondary care began to increase. Together these factors led to improved cooperation and a successful shift of stable diabetes patients from secondary to primary care.

### Decision support

#### Innovation

Three interviewees indicated that the reporting of protocol based care provision in the care chain information system allowed for performance monitoring, which, in combination with quality audits, helped to provide health care professionals with insights into their own performance and to compare them to their colleagues in the region. It also provided the basis for targeted plans for improvement and helped in demonstrating the positive outcomes achieved for diabetes care. This positive view was shared by the care purchasers who believed that performance monitoring increased the transparency and quality of care.

#### Organisational Context

As explained above, financial compensation is based on the health care professionals' adherence to the care group’s care protocols. On the one hand, this has led to clear guidelines for individual health care professionals as well as a clear division of tasks and responsibilities among several health care professionals cooperating around a specific patient. On the other hand, however, several interviewees thought that, due to the obligatory use of care protocols, some patients received more and others less care than necessary:

*“You are bound to a set of rules, which is good on the one hand, that you have certain guidelines to provide care, that they say: We expect you to do this for each patient every year. But given that the population is so diverse, not everyone needs the same kind of care and it has become more difficult to keep this personalised. What used to be easier before has changed now because I have to deliver certain outputs which are expected of me. And I think that some people receive more care than they actually need, but I also see other patients to whom I would have preferred to give more.* Care group B, podiatrist

Another interviewee explained how strictly protocol-based care provision led to a one-size-fits-all approach to care delivery, which in turn, stood in the way of high quality, person-centred care:

*“If you have a meat chopper and you put meat in it, it doesn’t matter what you put in it, you will always get minced meat. So in the past we might have had nothing, that’s true, so I think that the quality of care has improved. Back then maybe we had nothing, but now we turn everyone into minced meat. And that’s not fair to the patient because every patient gets the same standard product and very little profiling takes place because the financing and accounting culture and the current indicator-mania make this impossible”.* Care group A, specialist

### Clinical information system

#### Innovation

Interviewees indicated insufficient integration between the various electronic databases used by GPs, PNs, care chain partners and hospitals. As mentioned above, within the care group, GPs often use their own GP information system (HIS), practice nurses and care chain partners use the care chain information system (KIS), and care chain partners also use their own profession-specific databases. Hospitals also use their own system, which is separate from the systems used in primary care.

In care group A, not all HISs were integrated with the care group’s KIS, and hence, in these cases, PNs had to enter patient data twice. In care group B, the HIS and KIS were integrated to the extent that data entered in one system were automatically displayed in the other system. However, extracting data from the system for purposes of performance monitoring resulted in faulty or incomplete reports because the data originated from two different sources.

In both care groups, most care chain partners cooperated with multiple care groups in the same region, which meant that they had to work with several care chain information systems in addition to their own administrative system. One dietician explained that some of her colleagues worked with up to six different systems, especially when they also worked in the hospital. For PNs and care chain partners alike, this double data entry took longer, increased the likelihood of incorrect and incomplete data and led to staff dissatisfaction. Moreover, interviewees pointed out that sometimes the registration and data entry took up so much of the consultation time that it impeded on the time spent with the patient:

*“You have to enter things twice and that takes a lot of time. I think it’s a pity we spend a lot of time on IT now and registering things. Even though I understand that it needs to be done, it all needs to be transparent and clear, but because of this we sometimes have too little time to really provide care and I think… The patient is much more important to me… capturing data should be a minor matter but because you have two systems, well, it just takes a lot of time”.* Care group A, PN

Finally, when patients were referred to hospital, they disappeared from the primary care information systems until they returned to primary care, because the primary and secondary care information systems were incompatible. In those periods, the internist could access the care chain information system on a read-only basis.

#### Organisational context

Six interviewees pointed out the usefulness of the shared care chain information system, especially because it provided access to the patient’s electronic medical record to all involved health care professionals:

*“So the whole idea of cooperating around a patient and making sure that you do it in a coherent way has been helped a lot by the possibility to register everything in one place so that you could see and read what everyone registered”.* Care group B, director

Most notably, this meant that, at the first point of contact between a care chain partner and the patient, the health care professional had access to the patient’s electronic medical record, a source of information considerably richer than the paper-based referral or the patient’s memory. Moreover, it became clearer who the patient’s primary health care professional was and who bore responsibility for which aspects of the patient’s care.

## Discussion

The above presented an exploratory study on the implementation of integrated care for type 2 diabetes by two Dutch care groups, examining how the context and mechanisms of the intervention affected its outcomes.

Regarding mechanisms, our mapping of current core elements of Dutch integrated care to CCM components showed Dutch integrated care to reflect five of the six CCM components, namely health system (care groups and bundled payment system), self-management support (patient involvement), delivery system design (cooperation and substitution), decision support (evidence-based care protocols) and clinical information system (shared clinical information system). Aspects related to the community component were not found. The fact that integrated care touches upon almost all CCM components shows that many different aspects are being taken into account which is appropriate for a complex intervention. At the same time this means that efforts are divided over several focal points and therefore not implemented equally well in all areas. At the moment emphasis is put mostly on the health system, delivery system design, decision support and clinical information system. Self-management support is receiving much attention in the care standards but this has not been translated into the practice setting yet. Moreover, the fact that the community component has not yet been incorporated into the intervention underscores that integrated care is still in development.

Barriers were found at all levels of the IM and included insufficient integration between the various patient databases (innovation), decreased earnings for some health professionals (individual professional), patients’ insufficient medical and policy-making expertise (patient), resistance by GP assistants due to perceived competition (social context), too much care provided by PNs instead of GPs (organisational context) and the funding system incentivising the provision of care exactly as described in the care protocols (economic and political context). Facilitators were also found at all levels of the IM and included performance monitoring via the care chain information system (innovation), increased earnings for some health professionals (individual professional), increased focus on self-management (patient), innovators in primary and secondary care (social context), PNs and DNSs acting as integrators (organisational context) and financial incentives for guideline adherence (economic and political context). These findings show that the implementation of a complex intervention is in itself complex, too: various factors impact in different ways on different outcomes. The fact that the economic and political context as well as health IT-related barriers were discussed most frequently by the interviewees points towards those obstacles that are currently problematic. The fact that patient level barriers and/or facilitators have hardly been discussed at all points towards the need for increased focus on these stakeholders in practice as well as future research.

According to the interviewees, integrated care has led to perceived improvements in certain aspects of quality of care such as improved communication and cooperation but also to perceived deteriorations in others such as insufficient and unnecessary care provision and the preconditions for person-centred care. However, with so many diverse factors impacting on outcomes achieved, respondents’ opinions varied on whether, overall, integrated care had led to improved or deteriorated quality of care. This finding is in line with the findings of a large-scale evaluation of the bundled payment system three years after its introduction which also found small improvements to processes as well as some outcome indicators but concluded that it was not clear what these meant for overall quality of care [24]. A report on the status-quo of health IT use in Dutch integrated care also concluded that an overall health IT system has not been fully implemented yet and that often parts of the system are incompatible with each other and consequently cause problems in other areas as well [27].

There are several limitations associated with this study. First, there is no uniform way of describing or analysing the implementation of integrated care. Our interpretation of the CMO Model, defining mechanisms as the different aspects of the integrated care intervention, context as the barriers and facilitators encountered during the implementation, and outcomes as quality of care, is probably not the only way this could be done. However, we believe that this study’s approach, being embedded in three internationally accepted and widely-used conceptual models, is a valuable starting point for a more tangible way of evaluating integrated care interventions using the CMO approach.

Second, despite the importance attributed to the role of patients in integrated care, our study has found very little evidence on patient-level barriers and facilitators. This is probably at least partially due to the lack of patient interviews included in this research. However, it should be emphasised that all interviewees were specifically asked for their opinion about patient perspectives on integrated care. The lack of patient-related context information could therefore also be a sign of a limited focus on this issue by the health care professionals and thereby be a result in itself, reflecting the need for increased emphasis on the patient’s perspective. This would correspond to the findings of a study on the effects of care standards for vascular risk management which found self-management support and patient involvement to be lacking [28].

Third, being a single-case study design our study is case- and context-specific and cannot claim generalisability to other settings or conditions. Nevertheless, we think that the experiences and examples provided will help organisations wishing to implement integrated care interventions in their own settings. Above all, the added value lies in pointing out potential speed bumps as well as solutions and where these can be expected.

## Conclusions

Barriers and facilitators were found at all levels of the Implementation Model, with economic and political context and health IT-related barriers discussed most frequently by the interviewees. On the one hand, the implementation of integrated care led to improved communication and cooperation but on the other hand also to insufficient and unnecessary care provision and deteriorated preconditions for person-centred care. These findings show that Dutch integrated care is still in development and that its implementation has not realised its full potential yet. Future efforts should therefore focus on actively developing all areas of integrated care. However, the most problematic areas (such as financial and health IT issues) or those that have not received enough or any attention yet (such as patient and community involvement) seem to warrant the most urgent attention. To achieve generalisability, future research should also focus on the development of an integrated framework for analysing the implementation of integrated care interventions for people with chronic conditions, focusing on the different mechanisms by which and context in which these are implemented and explicitly linking those factors to the outcomes achieved.

## Abbreviations

CCM: Chronic Care Model
CMO Model: Context + Mechanism = Outcome Model
COPD: Chronic obstructive pulmonary disease
DNS: Diabetes nurse specialist
GP: General practitioner
HIS: General Practitioner Information System (*Dutch: huisartseninformatiesysteem*)
IM: Implementation Model
KIS: Care Chain Information System (*Dutch: keteninformatiesysteem*)
PN: Practice nurse
